# Control of Bacterial Sulfite Detoxification by Conserved and Species-Specific Regulatory Circuits

**DOI:** 10.3389/fmicb.2019.00960

**Published:** 2019-05-14

**Authors:** Yi Jie Chelsea Tan, Chengzhi Zhao, Marufa Nasreen, Leo O’Rourke, Rabeb Dhouib, Leah Roberts, Ying Wan, Scott A. Beatson, Ulrike Kappler

**Affiliations:** Centre for Metals in Biology, School of Chemistry and Molecular Biosciences, The University of Queensland, St. Lucia, QLD, Australia

**Keywords:** sulfite oxidation, gene expression, gene regulation, extracytoplasmic function sigma factor, microorganisms

## Abstract

Although sulfite, a by-product of the degradation of many sulfur compounds, is highly reactive and can cause damage to DNA, proteins and lipids, comparatively little is known about the regulation of sulfite-oxidizing enzyme (SOEs) expression. Here we have investigated the regulation of SOE-encoding genes in two species of α-Proteobacteria, *Sinorhizobium meliloti* and *Starkeya novella*, that degrade organo- and inorganic sulfur compounds, respectively, and contain unrelated types of SOEs that show different expression patterns. Our work revealed that in both cases, the molecular signal that triggers SOE gene expression is sulfite, and strong up-regulation depends on the presence of a sulfite-responsive, cognate Extracytoplasmic function (ECF) sigma factor, making sulfite oxidation a bacterial stress response. An additional RpoE1-like ECF sigma factor was also involved in the regulation, but was activated by different molecular signals, taurine (Sm) and tetrathionate (Sn), respectively, targeted different gene promoters, and also differed in the magnitude of the response generated. We therefore propose that RpoE1 is a secondary, species-specific regulator of SOE gene expression rather than a general, conserved regulatory circuit. Sulfite produced by major dissimilatory processes appeared to be the trigger for SOE gene expression in both species, as we were unable to find evidence for an increase of SOE activity in stationary growth phase. The basic regulation of bacterial sulfite oxidation by cognate ECF sigma factors is likely to be applicable to three groups of alpha and beta-Proteobacteria in which we identified similar SOE operon structures.

## Introduction

Sulfite is a highly reactive sulfur oxyanion that occurs in both pro- and eukaryotes as a by-product of sulfur compound degradation or external exposure to sulfite (Kappler and Enemark, 2015; Kappler and Schwarz, 2016). Free sulfite can damage proteins, DNA and lipids through formation of adducts, and sulfite-oxidizing enzymes (SOEs) are found in nearly all forms of life (Zhang et al., 2011; Kappler and Enemark, 2015; Kappler and Schwarz, 2016). Interestingly, although SOEs from vertebrates and plants have been shown to be detoxification mechanisms that protect cells from sulfur stress, in bacteria SOEs have mostly been described as elements of energy conserving pathways, e.g., during chemolithotrophy (Hänsch et al., 2007; Simon and Kroneck, 2013; Kappler and Schwarz, 2016).

Bacterial growth on organosulfur compounds and chemolithotrophic growth on inorganic sulfur compounds leads to the formation of significant amounts of sulfite, and the structural and functional diversity of bacterial sulfite dehydrogenases that carry out this process has been well established (Kappler, 2008, 2011; Kappler and Schwarz, 2016). In contrast, comparatively little is known about how expression of these enzymes is regulated, which is key to revealing their cellular and physiological roles.

While in some bacteria such as *Deinococcus radiodurans* SOE genes appear to be always highly expressed (D’errico et al., 2006), in many bacteria SOEs have complex regulatory patterns where upregulation usually occurs in the presence of metabolizable sulfur substrates, but activity may also undergo growth phase dependent induction and show varying levels of basal expression in different bacterial strains and species (Kappler et al., 2000, 2001; Wilson and Kappler, 2009; Bastiat et al., 2012).

In some bacteria an extracytoplasmic function (ECF) sigma factor/anti-sigma factor (ASF) pair is encoded directly upstream of the genes encoding SOEs and has been proposed to be involved in regulating SOE expression (Kappler et al., 2001; Wilson and Kappler, 2009; Bastiat et al., 2012). ECF sigma factor- based gene regulation is essentialy controlled by the ASF that sequesters the sigma factor in the absence of an activating signal. The involvement of an ECF sigma factor in SOE gene regulation has been confirmed in a recent study of *Sinorhizobium meliloti* strain GMI11495 (Bastiat et al., 2012). In *Sm. meliloti* GMI11495 the SOE-associated ECF sigma factor RpoE4 was induced during stationary growth phase and was identified as the key regulator of the *sorT* gene that encodes the *Sm. meliloti* SorT SOE (Wilson and Kappler, 2009; Bastiat et al., 2012). The work also uncovered that a second ECF sigma factor, RpoE1, plays a role in inducing *sorT* expression during growth on taurine, but not in the presence of thiosulfate, the second RpoE4-activating substrate that was identified. Based on these results a model was proposed where during stationary phase and during growth on taurine activation of *sorT* occurs through the action of both RpoE4 and RpoE1, while during growth on thiosulfate activation of *sorT* expression required only the action of RpoE4. The molecular signal sensed by RpoE4 was proposed to be sulfite, as sulfite accumulated in the cultures of strains carrying mutations in the *sorT* gene.

**Figure 1:**
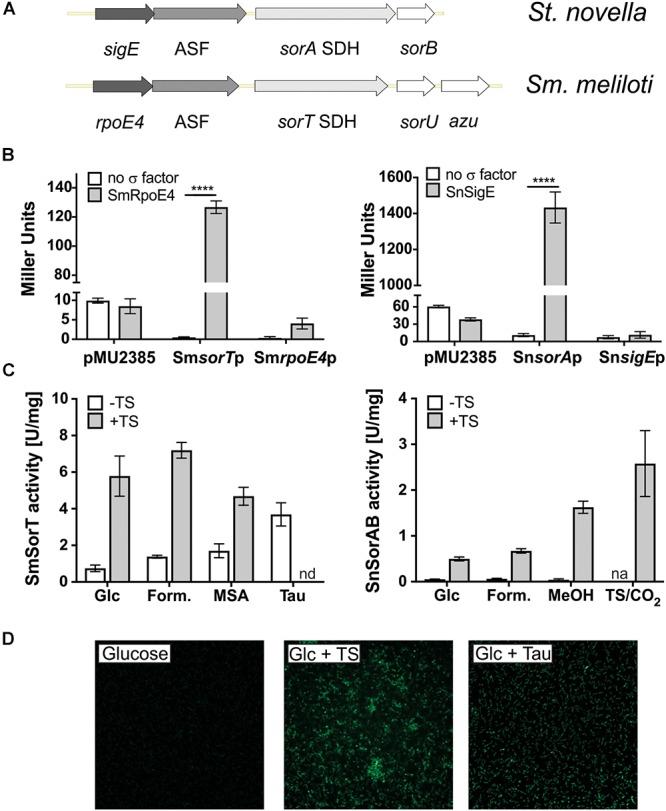
ECF sigma factor-based regulation of SOE gene expression in *Sm. meliloti* 1021 and *St. novella*. **(A)** Schematic representation of the *Sm. meliloti rpoE4/sorT* and *St. novella sigE/sorAB* gene regions, highlighting the similarity of the gene arrangement. **(B)** Reporter gene assays showing the ability of SmRpoE4 and SnSigE to activate the promoters of the cognate SOE encoding genes *sorT* and *sorA*. Controls shown test the activity of promoter gene fusions in the absence of a specific transcription factor (white columns) and the ability of the transcription factors to increase expression of the promoterless *lacZ* gene in pMu2385 (Label: pMu2385), respectively. Positive interactions between test promoters and sigma factors lead to increase beta-Galactosidase activity, 2-way ANOVA: ^∗∗∗∗^*p* < 0.0001. **(C)** Carbon and sulfur substrate dependent changes in SOE activity in *Sm. meliloti* 1021 and *St. novella*, highlighting the different modes of basal regulation. Enzyme activities were determined in cell extracts from cultures grown to late exponential growth phase on the indicated substrates using ferricyanide-based (*Sm. meliloti* SorT) or cytochrome *c*-based (*St. novella* SorAB) assays. The growth substrates chosen reflect the different substrate preferences of the bacteria. **(D)** *In vivo* induction of *sorT* promoter-*gfp mut2* fusions in *Sm. meliloti* growing on media with different growth substrates. Fluorescence changes correspond to SOE activity changes shown in **(C)**. Data in **(B,C)** represent averages of at least three repeat assays (*n* = 3 biological replicates), error bars represent standard deviation. Abbreviations: ASF, antisigma factor; Azu, azurin; cyt.c, cytochrome *c*; Form., formate; Glc,-glucose; MeOH, methanol; MSA, methanesulfonic acid; n.d., not determined; n.a., not applicable; Tau, taurine; TS, thiosulfate.

We have previously described the presence of genes encoding ECF sigma factors upstream of SOE encoding operons for two soil bacteria, the *Sinorhizobium meliloti* 1021 type strain and *Starkeya novella* (Figure 1; Kappler et al., 2001; Wilson and Kappler, 2009). Both of these bacteria are α-Proteobacteria of the order Rhizobiales, but belong to the families Rhizobiaceae and Xanthobacteraceae, respectively. *Sm. meliloti* and *St. novella* have the ability to degrade organosulfur compounds and inorganic sulfur compounds, contain distinct types of SOEs, the homodimeric SorT and the heterodimeric SorAB, (Kappler et al., 2000; Kappler and Bailey, 2005; Mcgrath et al., 2015), and also show different SOE regulatory patterns (Kappler et al., 2001; Wilson and Kappler, 2009; Low et al., 2011). While in *St. novella* SorAB SOE activity is nearly undetectable in the absence of degradable sulfur compounds (∼0.05 U/mg, Figure 1 and Supplementary Table S1), in *Sm. meliloti* 1021 SOE activity is always detectable at basal levels of 0.7–1 U/mg (Figure 1 and Supplementary Table S1; Kappler et al., 2001; Wilson and Kappler, 2009). This differs from both the SOE activity pattern in *St. novella* and the observations made for SOE gene expression in *Sm. meliloti* strain GMI11495 by Bastiat et al. (2012).

Here we have used *Sm. meliloti* 1021 and *St. novella* to investigate the conservation of SOE regulatory patterns across species, the signaling molecule(s) that trigger SOE expression, and to determine whether regulation of SOE expression by two ECF sigma factors is unique to *Sm. meliloti* strains. Our investigations show that up-regulation of genes encoding SOEs is triggered specifically by the presence of sulfite in both species, making sulfite oxidation a bacterial stress response and negating a major role in energy conservation. The main mode of SOE regulation in both species depended on the cognate ECF sigma factors SmRpoE4 and SnSigE. An additional, overlapping regulatory circuit depending on an RpoE1-like ECF sigma factor was also found in both species, but the respective RpoE1 sigma factors targetted different promoters within the SOE and ECF sigma factor operons (*Sm. meliloti rpoE4* promoter, *St. novella sorA* promoter). Additionally, RpoE1-based activation was based on species-specific stimuli and appeared to account only for a comparatively small fraction of SOE induction under very specific conditions. Phylogenetic analyses revealed at least three clades of ECF sigma factors found in association with SOE genes, one of which is unstudied to date and is comprised of species that appear to lack an RpoE1 homolog, making regulation by the cognate ECF sigma factor the main mode of SOE regulation.

## Materials and Methods

### Bacterial Strains, Plasmids, Media, and Growth Conditions

Bacterial strains and plasmids used in this study are listed in Supplementary Table S2. *E. coli* strains were routinely grown aerobically on Luria Bertani (LB) medium (Ausubel et al., 2005) at 37°C. *Starkeya novella* DSMZ506^T^ and *Sinorhizobium meliloti* strain 1021 were cultivated aerobically on either TYS medium (Beringer, 1974) or modified DSMZ medium no 69 at 30°C (Wilson and Kappler, 2009). The DSMZ 69 basal medium was supplemented with either 80 mM methanol, 20 or 40 mM thiosulfate, 20 mM tetrathionate, 10 mM glucose, 20 mM formate, 20 mM methanesulfonic acid, 20 mM taurine, or combinations of these compounds. Where applicable the following antibiotics were added to the growth media (μg/mL): *E. coli*: ampicillin and kanamycin 100, gentamicin and tetracycline 10, trimethoprim 30; *Sm. meliloti*: streptomycin 25, tetracycline 5, kanamycin 200.

### Molecular Biology Methods

Standard methods were used throughout (Ausubel et al., 2005). The PureLink Quick plasmid prep and PCR purification kits (Life Technologies) were used for the purification of plasmid DNA, PCR products and preparative restriction enzyme digests, restriction enzymes were from Life Technologies, T4 DNA ligase from Promega. GoTaq green Mastermix (Promega) was used for all standard PCR reactions, Pfu (Stratagene) or Phusion (Finnzymes) polymerases were used for all cloning applications and to generate probes for EMSA assays. Oligonucleotide primers (Supplementary Table S3) were from Life Technologies or IDT DNA technologies.

For gene expression experiments cultures were grown to early/mid–exponential growth phase on glucose or methanol containing medium 69 before sulfur compounds were added (20 mM taurine, 20 mM thiosulfate, or 1 mM sulfite). For SOE gene induction experiments samples for RNA isolation (2 mL) were taken just before addition of the compounds (*t* = 0) and at 15, 30, 60, 90, 120, and 180 min post-addition. All samples were preserved with RNAProtect Bacteria reagent (Qiagen), RNA was isolated using the Illustra RNAspin mini kit (GE Biosciences) and stored at -80°. gDNA was removed by DNAse treatment (TurboDNA free, Life Technologies), all samples were tested for the absence of gDNA using PCR amplification of the 16S rDNA gene. cDNA was prepared from 500 ng of DNA-free RNA using Superscript III or IV enzymes (Life Technologies) and random hexamer primers.

Quantitative RT-PCR used the SYBR green mastermix (Applied Biosystems) and was essentially carried out and analyzed as in Ang et al. (2017). The final reaction volume was 10 μL and 384 well plates were used throughout. An epMotion workstation (Eppendorf) was used to set up reactions, data was collected using a Quantstudio 6 (Life Technologies) thermal cycler. Gene expression was normalized to 16S gene expression, PCR efficiencies were determined using LinReg (Ramakers et al., 2003).

Transcription start sites were determined using the Life Technologies 5′RACE system according to the manufacturer’s instructions. DNA sequencing at the Australian Equine Genetics Research Centre (University of Queensland) used BigDye v3.1 (Applied Biosystems). EMSA experiments used the Dig-Gelshift Kit v2 (Roche Applied Science). EMSA reactions used a buffer containing 40 mM HEPES pH 7.9, 60 mM KCl, 1 mM MgCl_2_, 12% glycerol, and 1 mM DTT (Rhodes et al., 1997), RNA polymerase core (Epicenter), 0.25 pmoles DIG-labeled probe, 20 pmoles purified SnSigE, salmon sperm DNA or poly d[IC] (Sigma-Aldrich) were added where applicable. Binding reactions were incubated for 40 min at 28°C before being separated on a 5% 0.5× TBE acrylamide gel at 4°C followed by blotting and detection according to the manufacturer’s instructions.

### Creation of Gene Knock-Out Mutations

Gene knockout plasmids pKnock-Km-*rpoE4* and pKnock-Km-*sorT* were created by insertion of 300–400 bp gene fragments into the pKnock-Km vector (Alexeyev, 1999). Electrocompetent *Sm. meliloti* cells were prepared using 200 mL TYS-based cultures grown at 30°C, 200 rpm to an OD_600_ of ∼0.4–0.6. Cells were harvested by centrifugation (4000 × *g*, 4°C, 15 min), washed twice in 50 mL sterile water before resuspending in 1 mL of sterile 10% glycerol. To a 100 μL aliquot of these cells, 0.5–1 μg of plasmid DNA were added, followed by electroporation (2500 V, 25 μF, 400 Ω, 2 mm cuvette) using a Bio-RAD genepulser. Electroporated cells were taken up in 1 mL TYS and incubated at 30°C with shaking for 12 h before plating on selective media. *St. novella* is not amenable to genetic manipulation (Davidson and Summers, 1983; Davidson et al., 1985; Kappler et al., unpublished), precluding similar experiments with this bacterium.

### Biochemical Methods and Production of Recombinant Proteins

SDS-PAGE was performed as in Laemmli (1970). Small volume (2–3 mL) cell extracts for enzyme assays were prepared from cultures (10–20 mL) grown to mid/late exponential growth phase using BugBuster Mastermix (Novagen). SOE activity assays were carried out as in Kappler et al. (2000), Wilson and Kappler (2009) and Low et al. (2011) with ferricyanide or cytochrome *c* as electron acceptors for SOEs from *Sm. meliloti* and *St. novella*, respectively. Sulfite concentrations in growth media were determined using fuchsin as in Grant (1947). Pfu (Stratagene) and Phusion (Finnzymes) polymerases were used to amplify gene fragments with high fidelity, e.g., the Sn*sigE* and Sm*rpoE* genes and promoter regions for cloning into pQE30 (Qiagen, Sn*sigE*) or pProex HTB (Life Technologies, Sm*rpoE4*, Sm*rpoE1*, Sn*rpoE1*) or for cloning of promoter regions into pMU2385 or for use in EMSA experiments. All protein expression plasmids were tested for successful protein expression in small scale expression experiments. 6xHis SnSigE was expressed in DH5α at 30°C, using 1 mM IPTG followed by incubation for 4 h before harvesting. The recombinant protein was purified under native conditions using Ni-NTA resin (Qiagen) as per the manufacturer’s instructions or following refolding from inclusion bodies using the protocols of Burgess (1996) and Burgess (2009). Under non-denaturing conditions, 6xHis SnSigE co-purified with *E. coli* RNA Polymerase.

### Reporter Gene Assays

Beta-galactosidase activity present in *E. coli* cells carrying fusions of test promoters to the promoterless *lacZ* gene in pMU2385 (Praszkier et al., 1992) and a second plasmid expressing the relevant sigma factors was determined using the method of Kidd et al. (2005). The sigma factor expression plasmids were the same that were used for protein purification. Plasmids without inserts were used for control reactions that test either the activity of promoters in the absence of a transcription factor or the ability of a sigma factor to alter expression of the pMU2385 promoterless *lacZ* gene. Promoter fragments used were between 300 and 500 bp and located directly upstream of the coding regions. *E. coli* cultures for reporter gene assays were inoculated from overnight cultures into 5 mL of LB, grown at 37°C to an OD_600_ of ∼ 0.4–0.6. Cells were harvested and resuspended in 1xPBS before being used for enzyme assays (Kidd et al., 2005). All assays were repeated at least once and carried out in triplicates for each repeat, enzyme activities are reported in Miller units.

For *in vivo* monitoring of SOE promoter activities the same promoter fragments that were used in pMU2385 were cloned into pBluescript-*gfpmut2* followed by subcloning of the entire expression cassette into pRK415 (Ditta et al., 1985) for transfer into *Sm. meliloti* as described above. The *in vivo* activity of promoter gene fusions to *gfpmut2* was detected by either epifluorescence or confocal laser microscopy at the SCMB microscopy facility in cultures grown to mid-exponential phase on DSMZ medium 69 with or without the addition of a sulfur compound.

### Phylogenetic Analyses

Protein homologs of SnSigE and SmRpoE4 were identified using the BLAST algorithm (Altschul et al., 1997). Analysis of gene environments for ECF26 sigma factors used data available in public databases (e.g., GenBank)^1^, the Vector Nti Advance 11 program suite (Life Technologies) was used to display and compare the sequence data. The Mist database (v 2.0) (Ulrich and Zhulin, 2007) was used to confirm the detection of ECF sigma factors in the genomes of the model organisms, the ECF finder tool^2^ was employed to check the classifications of sigma factor sequences. The complete list of sigma factor sequences is available in Supplementary Table S4. Full length protein sequences were aligned ClustalW subsequently analyzed in MEGA 7.0 (Tamura et al., 2011), phylogenetic trees were constructed using Neighbor Joining (NJ), Minimum Evolution (ME), UPGMA, and Maximum Likelihood (ML) algorithms. All analyses assumed uniform rates of evolution of the amino acid sequences. NJ, ME, and UPGMA used a Poisson model of substitution and pairwise deletion of gaps/ missing data while ML analyses used the Jones- Taylor Thornton Model and partial deletion for gaps/ missing data. Robustness testing was carried out using the bootstrap method with 500 resampling cycles. Sequences belonging to ECF groups 12 and 15 as defined in Staron et al. (2009) were used as reference groups (Supplementary Table S4). All of these sequences were clearly located on separate branches outside of the three ECF groups analyzed (data not shown). The consensus ECF26 promotor profile was generated using the MEME suite of motif-based sequence analysis tools^3^ (Bailey et al., 2009). Specifically, glam2 was used to create the promoter consensus profile and logo, glam2scan was used to search the publicly available genome sequences for the presence of this profile, tomtom was used to compare this profile against the prodoric database of gene regulation (version 8.9) (Muench et al., 2003; Gupta et al., 2007; Frith et al., 2008).

## Results

### ECF Sigma Factor-Based Regulation of Sulfite Oxidation in *Sinorhizobium meliloti* 1021 and *Starkeya novella*

Basic characterization showed that in *St. novella* and *Sm. meliloti* 1021 the ECF sigma factor and cognate ASF encoding genes located upstream of the SOE encoding genes are co-transcribed (Supplementary Figure S1), and reporter gene assays (Figure 1) confirmed a specific induction of both the Sm_*sorT* and Sm_*rpoE4* promoters by the SmRpoE4 sigma factor (260 and 16 times induction, respectively), and the ability of the SnSigE sigma factor to bind to and induce expression of the Sn_*sorA* promoter (129 times induction) but not the Sn_*sigE* promoter (Figure 1 and Supplementary Figure S2).

Transcription start site mapping by 5′RACE revealed that in keeping with this observation, the Sn_*sigE* promoter lacks the consensus (-35 GGAAT, -10 CGTC) found upstream of the Sm*sorT*, Sn*sorA*, and Sm*rpoE4* genes (Supplementary Figure S1). This consensus essentially matches the one determined for the *sorT* and *rpoE4* genes of *Sm. meliloti* GMI11495 (Bastiat et al., 2012; Schlüter et al., 2013), however, we noticed that the spacing of the -10 and -35 elements differed by 1 bp between some promoters which could alter relative promoter strength (Supplementary Figure S1). Bioinformatic searches of the *St. novella* and *Sm. meliloti* 1021 genomes (Galibert et al., 2001; Kappler et al., 2012) did not reveal additional genes with a strong match to this consensus sequence.

As already indicated in the introduction, carbon and sulfur source dependent induction of SOE activity in both bacteria was limited to substrates that can give rise to sulfite either through abiotic processes in the medium (e.g., thiosulfate), or as a result of degradation by the bacteria (SM: taurine, SN: thiosulfate and tetrathionate) (Figure 1 and Supplementary Table S1). A Sm_*sorT* promoter GFP*mut2* fusion introduced into *Sm. meliloti* 1021 confirmed this *sorT* gene induction pattern *in vivo*, with GFP fluorescence being strongest for growth media containing taurine or both glucose and thiosulfate (Figure 1).

### Sulfite Is the Molecular Signal Inducing SOE Expression in Both *Sm. meliloti* and *St. novella*

**Figure 2:**
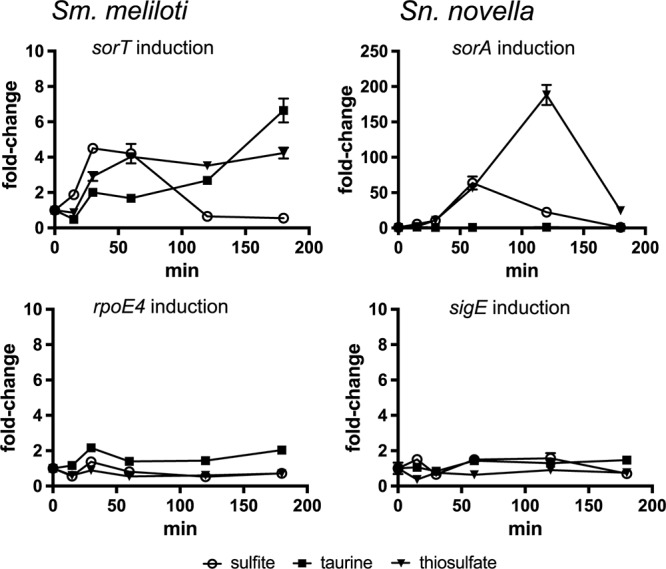
Fold-change in SOE and ECF sigma factor gene expression following exposure to sulfite, taurine and thiosulfate in *Sm. meliloti* 1021 and *St. novella* 506^T^. **Left:** *Sm. meliloti* gene expression induction, **Top**: induction of the *sorT* SOE gene, ***Bottom***: induction of the *rpoE4* ECF sigma factor gene. **Right:** *St. novella* gene expression induction, **Top**: induction of the *sorA* SOE gene, **Bottom**: induction of the *rpoE4* ECF sigma factor gene. Data are shown as the average and standard deviation of at least three assays. The underlying gene expression data is shown in Supplementary Figure S3. 2-Way ANOVA of changes in rel. normalized gene expression compared to the *t* = 0 value showed that for sulfite addition changes were statistically significant (alpha = 0.05) from *t* = 15 min (Sm) and *t* = 30 min (Sn) with *p* = 0.0032 (^∗∗^) (Sn, 30 min) to *p* < 0.0001 (^∗∗∗∗^) for all other values, for thiosulfate addition from *t* = 30 min (Sm) and *t* = 60 min (Sn) with *p* < 0.0001 (^∗∗∗∗^) for all datapoints, for taurine all values were not significant except Sm 120 min, *p* = 0.0232 (^∗^), and 180 min (*p* < 0.0001, ^∗∗∗∗^). For clarity the *p*-values are not shown in the figure.

The molecular signal sensed by the ECF type sigma factor/ASF pairs that regulate SOE gene expression could either be a metabolizable sulfur source, if induction were induced by degradation of a particular substrate, or sulfite itself if the main purpose of the SOE reaction is to detoxify sulfite. *Sm. meliloti* and *St. novella* cultures growing exponentially on glucose-containing media were exposed to taurine, thiosulfate or sulfite, which revealed an immediate, strong increase in Sm*sorT* and Sn*sorA* expression only in response to sulfite exposure (Figure 2 and Supplementary Figure S3). This increase in *sorA* and *sorT* gene expression was transient, not lasting beyond 2 h post-exposure, which is in keeping with the relatively low sulfite challenge concentration (1 mM) chosen to avoid toxicity and the rapid chemical and enzymatic turnover of sulfite in solution (Kappler et al., 2000; Wilson and Kappler, 2009).

Addition of thiosulfate also gave rise to an induction of Sm_*sorT/*Sn_*sorA* gene expression which, however, took about 60–120 min to peak, while taurine exposure caused Sm*sorT* gene expression to increase after >120 min incubation while no effect was observed in *St. novella* which is unable to metabolize taurine (Kappler et al., 2012; Figure 2). These observations are consistent with sulfite being the signal that induces SOE gene expression in both species. While *Sm. meliloti* is unable to metabolize thiosulfate, addition of thiosulfate to the DSMZ69 growth medium leads to the abiotic production of sulfite (∼120–160 μM) (Wilson and Kappler, 2009), explaining the thiosulfate-based induction of Sm_*sorT* gene expression in this species. In contrast, thiosulfate is also a major substrate for *St. novella* chemolithotrophic growth, and the *St. novella* thiosulfate degrading enzyme complex that leads to the formation of sulfite is always expressed at high levels (Kappler and Nouwens, 2013), allowing fast degradation of thiosulfate. This explains the strong upregulation of Sn_*sorA* gene expression observed in *St. novella* following addition of thiosulfate. In contrast, taurine degradation by *S. meliloti* that also gives rise to the formation of sulfite (Cook and Denger, 2002) is an inducible process, and would only produce sulfite following production of taurine-degrading enzymes and degradation of significant amounts of taurine, which is consistent with the observed lag of 120 min before induction for Sm_*sorT* was observed.

### Involvement of Multiple Regulatory Circuits in the Control of SOE Gene Expression Is Conserved Across Bacterial Species

Despite sulfite being the common molecular signal inducing SOE gene expression in *Sm. meliloti* and *St. novella*, with regards to the basal levels of activity the expression patterns for the SmSorT and SnSorAB SOEs differ clearly (Figure 1 and Supplementary Table S1), and one possible explanation for this could be that unlike what was found in *Sm. meliloti* (Bastiat et al., 2012), in *St. novella* only the cognate ECF sigma factor, SnSigE, might be involved in the regulation of SOE expression. To establish if regulation by two ECF sigma factors is present we first tested whether a homolog of the SmRpoE1 sigma factor exists in *St. novella*. The identified SnRpoE1-ASF pair is encoded by genes Snov_0992/0993 and SnRpoE1 is able to activate the promoter of Snov_0990 (Sn_*duf305*), a gene encoding a protein containing a domain of unknown function (DUF305), while a 270 bp region directly upstream of the Sn*rpoE1* gene (Snov_0992) showed no activation (not shown). The Snov_0990 (Sn_*duf305*) promoter likely also controls expression of the Snov_0991 gene that encodes a YVTN-beta propeller repeat protein from which the Snov_0990 gene is only separated by 12 bp.

**Figure 3:**
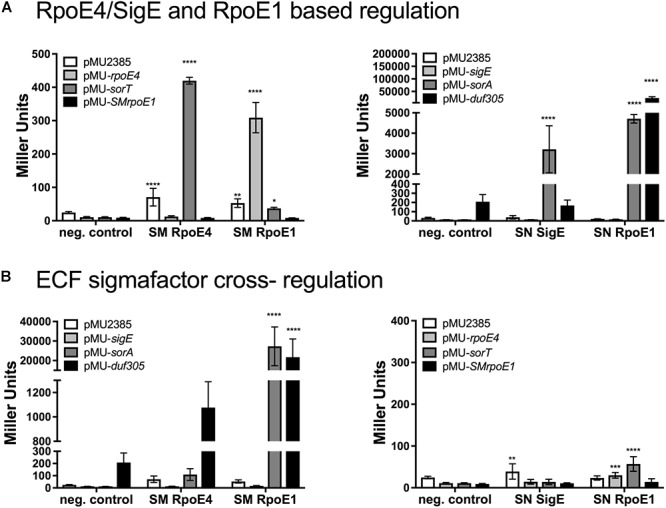
Reporter gene assays investigating promoter activation by *Sm. meliloti* and *St. novella* ECF sigma factors SmRpoE4/SnSigE and Sm/SnRpoE1. **(A)** *Sm. meliloti* RpoE4 and RpoE1activation of *Sm. meliloti rpoE4*, *sorT*, *rpoE1* promoters **(left)** and *St. novella* SigE and SnRpoE1 activation of *St. novella sigE*, *sorA* and *duf305* promoters **(right)**. **(B)** SmRpoE4/SnSigE and Sm/SnRpoE1-mediated regulation of SOE gene and ECF sigma factor promoters species. Activation of *St. novella* promoters *by Sm. meliloti* ECF sigma factors **(left)**, and activation of *Sm. meliloti* promoters by *St. novella* ECF sigma factors **(right)**. Data are shown as the averages and standard deviation of at least three assays. Statistical analyses used 2-Way ANOVA comparing data for each promoter to the negative control (no sigma factor present). ^∗∗∗∗^*p* < 0.0001, ^∗∗∗^*p* = 0.0006; ^∗∗^*p* = 0.0093; ^∗^*p* = 0.0106.

In reporter gene assays SnRpoE1 induced strong expression of the Sn_*duf305* and Sn_*sorA* promoters but not Sn_*sigE*p, while SnSigE strongly induced Sn_*sorA*p and also showed a weak ability to induce Sn_*duf305*p activity (Figure 3).

Matching experiments with SmRpoE1 revealed strong induction of the Sm_*rpoE4* promoter and very low level induction of the Sm_*sorT* promoter, while only SmRpoE4 showed strong induction of the Sm_*sorT* promoter (Figure 3).

Interestingly, the *Sm. meliloti* RpoE1 and RpoE4 sigma factors were also able to interact with the three *St. novella* promoters tested here (Figure 3). SmRpoE4 induced low level Sn_*sorA* promoter activity and medium level expression of the Sn_*duf305* promoter, while SmRpoE1, similar to SnRpoE1, gave rise to high levels of Sn_*sorA*p and Sn_*duf305*p activation, while no changes in Sn_*sigE*p activity were observed.

In contrast, SnSigE and SnRpoE1 were unable to recognize the *Sm. meliloti sorT, rpoE4*, or *rpoE1* gene promoters (Figure 3). The ability of SmRpoE1 for interspecies cross-regulation was confirmed using an Sn_*sorA* promoter-GFP*mut2* fusion introduced into *Sm. meliloti*, where even on media containing only glucose, i.e., non-inducing conditions, strong fluorescence was observed (Supplementary Figure S4). The constant activity of the Sn_*sorA* promoter in *Sm. meliloti* 1021 (Figure 3 and Supplementary Figure S4), suggests that in this strain SmRpoE1 was active under all conditions tested.

### SnRpoE1 Likely Controls a Second, Tetrathionate-Responsive Regulatory Circuit That May Be Able to Activate Sn*sorAB* Expression Under Specific Growth Conditions

**Figure 4:**
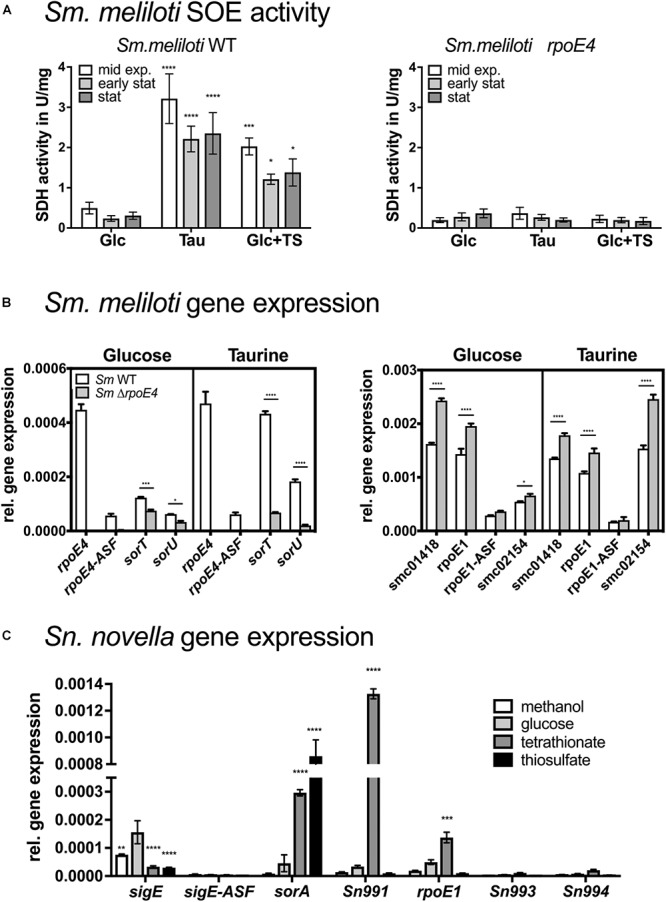
SOE activities and expression of SOE and ECF sigma factor-associated genes in *Sm. meliloti*^WT^ and *Sm. meliloti*^Δ^^*rpoE*^^4^ strains **(A,B)** and in *St. novella*^WT^ **(C)**. **(A)** Growth- phase dependent changes in SOE activity in *Sm. meliloti*^WT^ and *Sm. meliloti*^Δ^^*rpoE*^^4^ on glucose, taurine and glucose and thiosulfate. **(B)** Expression of SmRpoE4 and SmRpoE1 associated genes in *Sm. meliloti*^WT^ (white) *Sm. meliloti*^Δ^^*rpoE*^^4^ (gray) during growth on glucose or taurine. **(C)** Expression of genes associated with the SnSigE and SnRpoE1 encoding genes in *St. novella*^WT^ under different growth conditions. Abbreviations: Glc, glucose; TS, thiosulfate; TT, tetrathionate. Growth with TS and TT as substrate was under chemolithoautotrophic conditions. All data are shown as averages and standard deviations of at least three replicate determinations. 2-Way ANOVA was used to analyse data. ^∗∗∗∗^*p* < 0.0001, ^∗∗∗^*p* = 0.0002–0.0005, ^∗∗^*p* = 0.013, ^∗^*p* = 0.0101–0.0362.

We then analyzed expression of the SOE, sigma factor and ASF genes in *St. novella* grown on glucose (heterotrophic growth), methanol (C1-compound growth), thiosulfate and tetrathionate (both chemolithoautotrophic growth). The gene expression data confirmed the strong induction of *sorA* gene expression in the presence of thiosulfate (∼18 times rel. to glucose levels) also reflected in the enzyme activities (Figure 1, 4). Both Sn*sigE* and Sn*rpoE1* genes were expressed at similar levels, with sigma factor gene expression levels significantly exceeding (∼10×) those of the respective cognate ASF (SnSigE ASF: Snov_3267, SnRpoE1 ASF: Snov_0993). Expression of Sn*rpoE1* and associated genes (Snov_0990-0993) was low in glucose, methanol and thiosulfate samples, indicating that RpoE1 was probably not active under these conditions. However, during growth on tetrathionate, activation of SnRpoE1 occurred as indicated by high levels of expression of the Snov_0991 gene that is associated with the gene encoding the Snov_0990 DUF305 protein used in promoter studied (Figure 4). As reporter gene assays with SnRpoE1 showed strong interactions with the Sn_*sorA* promoter, this suggests that *in vivo* during growth on tetrathionate SnRpoE1 may have activated Sn_*sorA* transcription. However, a contribution of the sulfite-responsive SnSigE sigma factor to Sn_*sorA* expression on tetrathionate cannot be excluded as the degradation pathway for tetrathionate in *St. novella* is unknown and may lead to the formation of sulfite which would trigger the SnSigE response. This then suggests that the regulatory pattern of the *St. novella* SorAB SOE depends mostly on the activation of the cognate SnSigE sigma factor.

### Mutations in Sm*rpoE4* or Sm*sorT* Do Not Alter Growth Phenotypes and Do Not Reveal Growth Phase Dependent Activation of SmRpoE4

The constant activation of the RpoE1 ECF sigma factor in *Sm. meliloti* 1021 indicated by the constant activation of the Sn_*sorA*p (Supplementary Figure S4) is likely to impact expression patterns of *sorT* and the *rpoE*4 associated genes, and we assessed this using *Sm. meliloti* 1021 *sorT* or *rpoE4* gene knockout strains. All strains showed similar growth rates, with growth on taurine being slower than on glucose- containing media, on which, interestingly, the *Sm. meliloti*^ΔrpoE4^ strain showed slightly reduced growth (*p* < 0.01) (Table 1 and Supplementary Figure S2), although this was not the case in the presence of taurine.

**Table 1:** Growth rates of *S. melilot* WT and mutant strains under different conditions.

	Growth Rates (h^-1^)		
Strain	Glucose	Taurine	Glucose + Thiosulfate
*Sm. meliloti* 1021 WT	0.232 ± 0.005	0.116 ± 0.003	0.232 ± 0.003
*Sm. meliloti* 1021 Δ*rpoE4*	0.195 ± 0.003	0.124 ± 0.003	0.200 ± 0.003
*Sm. meliloti* 1021 Δ*sorT*	0.223 ± 0.002	0.111 ± 0.001	0.213 ± 0.004

*Values present average growth rates and standard deviations determined for at least three biological replicates per strain and growth condition*.

SorT enzyme activity levels in *Sm. meliloti* 1021^WT^ were as expected, with low activities for glucose-containing media and high activity when taurine or glucose and thiosulfate were used as substrates (Figure 4 and Supplementary Figure S5). In contrast, only basal SorT activity levels of ∼0.2–0.3 U/mg were present at all times in the *Sm. meliloti*^ΔrpoE4^ strain, while in the *Sm. meliloti*^Δ^^*sorT*^ strain, as expected, no activity was detected. Under all conditions tested, SmSorT SOE activity was maximal during exponential growth, while in stationary phase SorT activity levels were reduced by 20–40% relative to exponential growth (Figure 4 and Supplementary Figure S5). This was also true for glucose-containing medium, where a stationary-phase dependent induction due to increased internal sulfite formation should have been most apparent as a result of the absence of a growth substrate generating sulfite.

The basal activity observed in the *rpoE4* knockout strain likely resulted from the weak interaction of SmRpoE1 with the Sm_*sorT* promoter observed in reporter gene assays. The comparatively higher basal levels of *sorT* expression in *Sm. meliloti* 1021^WT^ (0.7–1.0 U/mg, Supplementary Table S1) is likely due to activation of the Sm_*rpoE4* promoter by SmRpoE1, which was seen in the *in vitro* experiments.

Gene expression analysis in *Sm. meliloti* 1021^WT^ and *Sm. meliloti*^Δ^^*rpoE*^^4^ strains matched expectations for the *rpoE4* and *sorT* operons. Maximal Sm*sorT* gene expression occurred in the presence of thiosulfate, while during growth on glucose only low levels of Sm*sorT* expression were observed (Figure 4). Sm*rpoE4* and the cognate antisigma factor gene *SMc_04050* were expressed at low but near constant levels regardless of the growth medium with ASF expression reduced to ∼12–17% of Sm*rpoE4* expression levels (Figure 4 and Supplementary Figure S6).

The same pattern of expression was also seen for Sm*rpoE1* and *SMc_01420* (RpoE1 ASF) where despite overall very high levels of gene expression under all conditions, expression of the ASF was reduced by ∼80–85%, while expression of the associated *SMc_01418* gene was high throughout and showed very little variation with growth conditions (Figure 4).

For the Sm*rpoE4* knockout strain expression patterns for genes in the Sm*rpoE1* gene region were unaffected, while expression of Sm*rpoE4* and *SMc_04050* was absent. Expression of Sm*sorT* was reduced to basal levels confirming the results from enzyme assays (Figure 4 and Supplementary Figures S5, S6).

Of the additional genes proposed to be part of the SmRpoE1 regulon by Bastiat et al. (2012) only SMc_02154 showed significant expression, with expression levels and patterns being similar to those seen for Sm*rpoE1* itself. In contrast, the genes proposed to be controlled by SmRpoE4 (*SMc04164*, *SMc00821*, and *SMc00108*) were expressed at very low levels throughout and showed no variation between the *Sm. meliloti*^WT^ and *Sm. meliloti*^Δ^^*rpoE*^^4^ strains, indicating that they are not subject to direct regulation by SmRpoE4 in *Sm. meliloti* 1021 (Figure 4 and Supplementary Figure S6).

## Discussion

Sulfite oxidation is an essential process in all bacteria that degrade sulfur compounds as either carbon or energy sources, and here we have shown that despite clearly differing types of sulfur metabolism, the presence of structurally distinct SOEs and differing patterns of SOE basal regulation, the basic mechanism of SOE regulation is conserved between two different species of soil bacteria, *Sm. meliloti* and *St. novella* (Kappler and Bailey, 2005; Mcgrath et al., 2015).

**Figure 5:**
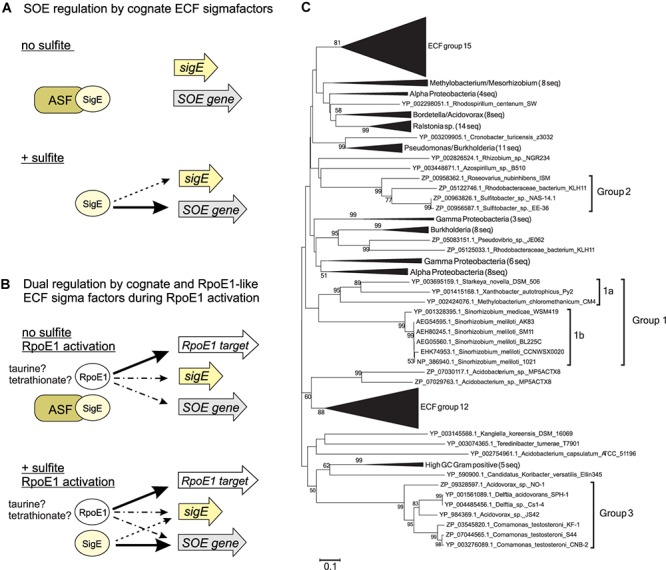
Model of SOE gene regulation **(A,B)** and phylogenetic tree showing different groups of ECF sigma factors associated with SOE-encoding genes **(C)**. **(A)** Conserved mode of regulation of SOE gene expression by cognate ECF sigma factors. **(B)** Regulation of SOE gene expression by two ECF sigma factor regulatory circuits – magnitude of response and induction signal appear to be species-specific. **(C)** Neighbor -joining tree of ECF sigma factors belonging to ECF group 26 with ECF groups 12 and 15 used reference as groups. The three groups of SOE associated ECF sigma factors are indicated by thicker lines and labels, *St. novella* SigE and *Sm. meliloti* RpoE4 are highlighted through use of a larger font and underlining. Bootstrap values are given as percent conservation over 500 replications. Abbreviations: ASF, antisigma factor; SigE, SOE-gene associated ECF sigma factor; SOE gene, gene encoding a sulfite oxidizing enzyme; RpoE1 target, gene controlled by RpoE1 ECF sigma factor; solid arrows, indicate positive regulation with arrow thickness indicating level of activation; dotted arrows/dot and dash arrows, proposed interactions or interactions that were not consistent between the organisms studied here. Dotted lines: Interaction of SOE cognate sigma factors with their own promoters which are not always present; dots and dashes: RpoE1 based activation of SOE associated genes, not all of them are realized in each species studied here (Supplementary Figure S8).

In both species induction of SOE gene expression is triggered by sulfite, and depends mostly on the respective, cognate ECF sigma factor. With sulfite as the trigger, induction of SOE expression in both species is specific to substrates that lead to sulfite formation either via abiotic processes (e.g., thiosulfate) or are metabolized with sulfite as an intermediate (Sn: thiosulfate and possibly tetrathionate, Sm: taurine). Induction of SOE gene expression was tightly linked to the presence of sulfite produced externally or as a result of energy conserving metabolic processes during exponential growth, and we found no evidence for significant SOE gene expression during stationary growth phase as a result of increased internal digestion processes. We therefore propose a simplified model for SOE activation that is likely to be applicable to many bacterial species where ECF sigma factors are found up- or downstream of SOE encoding genes, and where regulation depends essentially on the presence of sulfite which leads to release of the cognate ECF sigma factors from their respective ASFs by an as yet unknown mechanism (Figure 5 and Supplementary Figures S7, S10).

An additional layer of complexity can be added to SOE gene regulation by the presence of a second regulatory circuit that involves RpoE1-type ECF sigma factors. RpoE1 sigma factors from both *Sm. meliloti* and *St. novella* were able to interact with either the promoters of the SOE encoding genes and/ or the promoters of the cognate ECF sigma factors (SmRpoE4/SnSigE) *in vitro*, but the interaction strength and promoter preferences differed clearly between species (Figure 3). While SnRpoE1 strongly activated the Sn_*sorA* promoter, but did not interact with the Sn_*sigE* promoter, SmRpoE1 was able to primarily activate Sm_*rpoE4*p and, to a much lower extent, the Sm_*sorT* promoter. This indicates that in *St. novella*, where this sigma factor has never been studied, RpoE1 might exert its effect by directly activating the Sn_*sorA* promoter, while in *Sm. meliloti* RpoE1-based regulation would occur mainly via activation of Sm*rpoE4* expression. SnRpoE1 may thus be responsible for activation of Sn*sorA* gene expression when the SnRpoE1 activating substrate, tetrathionate, is present in the growth medium.

In *Sm. meliloti* strain 1021 SmRpoE1 appeared to be constantly activated as well as overexpressed (Figure 1, 4 and Supplementary Figure S4), confirming that the C-terminal truncation of the SnRpoE1 ASF encoding gene (SMc_01420) discovered in earlier studies abolishes its ability to effectively sequester RpoE1 (Schlüter et al., 2013). However, the constant activation of SmRpoE1 clearly demonstrated that SMRpoE1 is able to activate Sm*sorT* gene expression *in vivo*, and explains the unusual, high basal SOE activity present in this strain (Figure 1). In *Sm. meliloti* GMI11495 where, as in most *Sm. meliloti* strains, the SmRpoE1 ASF is not truncated, RpoE1-based Sm*sorT* gene expression was only observed in the presence of taurine (Bastiat et al., 2012).

Overall, while RpoE1 mediated regulation of SOE gene expression is present in both species, the specific conditions under which this regulatory circuit is required vary significantly, as does the magnitude and mode of SOE gene regulation in the two species. We therefore propose that RpoE1 based regulation is a secondary mechanism of SOE gene regulation that will have to be studied in the context of sulfur metabolism present in species that contain both RpoE4/SigE-like and RpoE1-like ECF sigma factors (Figure 5 and Supplementary Figure S7).

In summary we have here presented an updated model of activation of SOE gene expression by ECF sigma factors that shows that the presence of sulfite is the main driver of SOE gene expression in two species of α-Proteobacteria that represent different types of bacterial sulfur metabolism, and that co-regulation of SOE gene expression by a second ECF sigma factor occurs in both species but uses distinct mechanisms of regulation. ECF sigma factor genes found in close proximity to SOE encoding genes occur in at least three phylogenetically distinct bacterial groups, two of which contain α-proteobacterial species (group 1 – general and plant-associated soil bacteria such as Rhodobacterales and Rhizobiales, group 2 – mostly marine Rhodobacterales), while the third one exclusively contains β-proteobacterial species (group 3 – mostly Burkholderiales sequences, group 3a *Delftia* sp., *Comamonas* sp. and *Bordetella* sp., group 3b: *Acidovorax* sp., *Thauera* sp., and *Burkholderia* sp.; total of 130 sequences in all three groups) (Figure 5 and Supplementary Figures S8, S9). The promoter consensus sequence (-35 GGAAT and -10 CGTC) (Supplementary Figure S1) identified in *Sm. meliloti* and *St. novella* (representatives of groups 1a and 1b, respectively) was present upstream of SOE-encoding genes from phylogenetic groups 1 and 2 (both α-Proteobacterial clusters). There is a strong similarity between the predicted consensus for the SmRpoE1 sigma factor (Schlüter et al., 2013) and the promoter consensus found upstream of the SOE-encoding genes, which may explain the observed co-regulation. This promoter consensus sequence did not appear to be present upstream of any of the group 3 SOE gene sequences and the three groups can also be distinguished based on the positioning of the ECF sigma factor encoding gene relative to the SOE encoding gene (Supplementary Figure S10).

Our phylogenetic analyses further suggest that the recruitment of the ECF sigma factors to SOE gene regions may have occurred at least twice, as the group 3 ECF sigma factors are only distantly related to those found in the α-Proteobacterial groups 1 and 2 investigated here (∼40% amino acid identity), and preliminary investigations also indicate that no close homolog of the RpoE1 ECF sigma factors exists in group 3 species. Given the large number of known ECF sigma factor groups (Staron et al., 2009) it is likely that additional groups of SOE associated ECF sigma factors exist, and future research should focus on these as well as SOE regulation in bacteria known to harbor SOEs not associated with ECF sigma factors. Further work is also needed to understand the molecular details of sulfite sensing by ASFs and release/activation of the sequestered sigma factor, and regulation of ECF sigma factors such as SnSigE that are not subject to autoregulation.

## Author Contributions

YT, CZ, MN, LO’R, RD, LR, and YW carried out the experimental work. SB contributed to the bioinformatic analyses. YT, RD, and LO contributed to the writing of the manuscript. UK supervised student experiments and was responsible for the preparation of the final manuscript and figures.

## Conflict of Interest Statement

The authors declare that the research was conducted in the absence of any commercial or financial relationships that could be construed as a potential conflict of interest.

## Footnotes

1 www.ncbi.nlm.nih.gov

2 http://ecf.g2l.bio.uni-goettingen.de:8080/ECFfinder/

3 http://meme.nbcr.net/
