# Supplementary material for: Control of Bacterial Sulfite Detoxification by Conserved and Species-Specific Regulatory Circuits
Source: Front Microbiol. 2019 May 14;10:960. doi: 10.3389/fmicb.2019.00960 (PMC6527743; doi:10.3389/fmicb.2019.00960)
Supplement: Supplementary file 2 [file Data_Sheet_1.PDF]

Supplementary figures and tables

ECF-sigma factor mediated responses to sulfite stress in soil bacteria

Yi Jie Chelsea Tan[1], Chengzhi Zhao[1], Marufa Nasreen[1], Leo O'Rourke[1], Rabeb Dhouib[1], Leah Roberts[1], Ying Wan[1], Scott A. Beatson[1], Ulrike Kappler[1]*

[1]School of Chemistry and Molecular Biosciences, Centre for Metals in Biology, The University of Queensland, St. Lucia Qld 4072, Australia.

Table S1 Induction of sulfite dehydrogenase (SOE) activity in *Starkeya novella* and *Sinorhizobium meliloti* in the presence of various carbon and sulfur sources. Thiosulfate (Sn) and taurine (Sm) containing media were used as a control for SOE activity in the two organisms, respectively. Assays were done in triplicate on cell-free extracts from three biological replicates for each condition, errors shown are standard deviations of the mean.

**Organism**	***Starkeya novella***		***Sinorhizobium meliloti***	
**SOE activity** (mU/mg)		**+ thiosulfate**		**+ thiosulfate**
Glucose	50 ± 4	496 ± 42	745 ± 180	5183 ± 1098
Formate	59 ± 12	672 ± 48	1379 ± 77	7193 ± 427
Methanol	33 ± 25	1625 ± 133	n.d.	n.d.
MSA	n.d.	n.d.	1703 ± 377	4683 ± 489
Taurine	n.d.	n.d.	3690 ± 635	n.d.
Thiosulfate/ CO_2	n.d.	2579 ± 719	n.d.	n.d.

n.d. – not determined, MSA- methanesulfonic acid

Table S2 – List of bacterial strains and plasmids used in this study

Strain or plasmid	Genotype or phenotype	source
Escherichia coli		
DH5α	F$^-$Φ80*lacZ*ΔM15 *end*A1*hsd*R17 (r_K^- m_K^-) *sup*E44 *thi*$^{-1}$*gyr*A96 Δ(*lacZYA-argF*)	Invitrogen
JM109 λpir	F′ *traD36 proA*$^+$*B*$^+$ *lacI*^{q} *Δ(lacZ)M15/ Δ(lac-proAB) glnV44 e14*$^-$ *gyrA96 recA1 relA1 endA1 thi hsdR17 λpir*	New England Biolabs
S17-1	*recA pro* (RP4-2Tet::Mu Kan::Tn7)	(Simon*, et al.*, 1983)
***Sinorhizobium meliloti* 1021**	Wild type, Type strain	(Galibert*, et al.*, 2001)
S.meliloti ΔsorT	*S. meliloti* 1021 *sorT*::kan	This work
S.meliloti ΔrpoE4	*S. meliloti* 1021 *rpoE4*::kan	This work
S.meliloti pRK-GFP*sorA*	*S. meliloti* 1021 with pRK-GFPsorA	This work
S.meliloti pRK-GFP*sorT*	*S. meliloti* 1021 with pRK-GFPsorT	This work
S.meliloti pRK415	*S. meliloti* 1021 with pRK415	This work
Starkeya novella **DSMZ 506^T**	Wild type, Type strain	DSMZ
**plasmids**		
**RACE plasmids**		
pGemT easy	PCR cloning vector, Amp^r	Promega
pGemT-sorA	pGemT with *S.novella sorA* RACE PCR product	This work
pGemT-sigE	pGemT with *S.novella sigE* RACE PCR product	This work
pGemT-sorT	pGemT with *S.meliloti sorT* RACE PCR product	This work
pGemT-rpoE	pGemT with *S.meliloti rpoE4* RACE PCR product	This work
**Gene knock-out plasmids**		
pKnock-Km	Gene knockout plasmid, Km^r	(Alexevey, 1999)
pKnock-Km-*sorT*	pKnock-Km with internal fragment (~ 400 bp) of *S. meliloti sorT* gene Km^r	This work
pKnock-Km-*rpoE4*	pKnock-Km with internal fragment (~ 300 bp) of *S. meliloti rpoE4* gene Km^r	This work
**ECF sigmafactor expression plasmids**		
pQE30	Protein expression plasmid, N-terminal His-Tag, Amp^r	Qiagen

pQE30-sigE	pQE30 with *S.novella sigE* gene, Amp^r	This work
pQE30-sigEV140P	pQE30 with *S.novella sigE* gene with V140P mutation, Amp^r	This work
pProex-Htb	Protein expression plasmid, N-terminal His Tag, Amp^r	Invitrogen
pProex-rpoE	pProex Htb with *S. meliloti rpoE4* gene, Amp^r	This work
pProex-SMrpoE1	pProex Htb with *S. meliloti rpoE1* gene, Amp^r	This work
pProex-SNrpoE1	pProex Htb with *S. novella rpoE1* gene, Amp^r	This work
**LacZ reporter gene assays**		
pMU2385	Reporter gene plasmid with promoterless lacZ gene, Tp^r	(Praszkier*, et al.*, 1992)
pMu2385-sorA	pMu2385 with *S.novella sorA* promoter, Tp^r	This work
pMu2385-sigE	pMu2385 with *S.novella sigE* promoter, Tp^r	This work
pMu2385-duf305	pMu2385 with *S.novella* duf305 promoter (Snov0990 gene), Tp^r	This work
pMu2385-sorT	pMu2385 with *S.meliloti sorT* promoter, Tp^r	This work
pMU2385-rpoE	pMu2385 with *S.meliloti rpoE4* promoter, Tp^r	This work
pMU2385-rpoE1	pMu2385 with *S.meliloti rpoE1* promoter, Tp^r	This work
**GFP-mut2 reporter gene assays**		
pBluescript II	Cloning vector, Amp^r	Stratagene
pDS439	Gfp mut2 genes under control of araBAD promoter	(Siegele & Hu, 1997)
pBluescript GFP-mut2	pBluescript with *gfp mut2* gene, Amp^r	This work
pRK415	Broad host range plasmid, low copy, Tc^r	(Ditta*, et al.*, 1985)
pRK-GFPsorA	pRK415 with *S. novella sorA* promoter fused to GFPmut2	This work
pRK-GFPsorT	pRK415 with *S. meliloti sorT* promoter fused to GFPmut2	This work

Alexeyev, M. F. (1999). The pKNOCK series of broad-host-range mobilizable suicide vectors for gene knockout and targeted DNA insertion into the chromosome of Gram-negative bacteria. *BioTechniques, 26*(5), 824-826.

Ditta G, Schmidhauser T, Yakobson E*, et al.* (1985) Plasmids related to the broad host range vector, pRK290, useful for gene cloning and for monitoring gene expression. *Plasmid* **13**: 149-153.

Galibert F, Finan TM, Long SR*, et al.* (2001) The composite genome of the legume symbiont *Sinorhizobium meliloti Science* **293**: 668-672.

Praszkier J, Wilson IW & Pittard AJ (1992) Mutations affecting translational coupling between the *rep* genes of an IncB miniplasmid. *Journal of Bacteriology* **174**: 2376-2383.

Siegele DA & Hu JC (1997) Gene expression from plasmids containing the araBAD promoter at subsaturating inducer concentrations represents mixed populations. *Proceedings of the National Academy of Sciences of the United States of America* **94**: 8168-8172.

Simon R, Priefer U & Puhler A (1983) A broad-host range mobilization system for in vivo engineering: transposon mutagenesis in Gram-negative bacteria. *Bio/Technology* **1**: 784-791.

Table S3 – List of oligonucleotide primers used in this study

**Promoter cloning into pMU2385**	
SMsorT_proF_Eco	AAAA GAATTC TTCCGGCAGCAACGACAGCT
SMsorT_proR_Bam	AAAA GGATCC TGAGCCTGTTCCGGATCGCT
SMsigE_proF_Eco	AAAA GAATTC CGAAAAGCCGCGCATAAT
SMsigE_proR_Bam	AAAA GGATCC TGGCGACAGGCGTCTGAAAC
SN_sorpr1B	AAAA GGATCC AGCGGAGGACGAGCGGAT
SN_sorprr1E	AAAA GAATTC GATTTGGCGTCTGTTGAG
SN_sigprr1B	AAAA GGATCC TCTCTCTCCGCGCTCGG
SN_sigprf1E	AAAA GAATTC TCGCTCCCTCTGCCATGG
**Generation of gene probes for EMSA**	
SN_sorA_SORPR1	AGCGGAGGACGAGCGGAT
SN_sorA_SORPRR1	GATTTGGCGTCTGAG
SN_sigE_SIGPRR1	TCTCTCTCCGCGCTGG
SN_sigE_SIGPRF1	TCGCTCCCTCTGCCATGG
**Cloning of GFPmut2 into pBluescript**	
GFPmut2F-BamHI	AAAA GGATCC ATGAGTAAAGGAGAAGAACT
GFPmut2R-HindIII	AAA AAGCTT CTTATTTGTATAGTTCATCCATG
**Primers for cloning of sigE/rpoE into expression vectors**	
SnSIG-EXF2	AAAGGATCCCGCGCCGCGATCGAAGT
SnSIG-EXR1	CATAAGCTTTCATTTTTCGCCATGCCG
SM_rpoEf_bam	AAAAGGATCCTCGGGTATCATGCGCTCGCG
SM_rpoEr_pst	AAAACTGCAGTCATTTTCGGCCTTCTCGATG
**Primers for generation of pKnock plasmids**	
SMsorT_kn_fwd	GGAAAGAGGCCTCCCGTTCATTT
SMsorT_kn_rev	CAACACGCCTCCGGAATCGATT
SNsorA_kn_fwd	AGGCGCCGTTCTCCACATTGGT
SNsorA_kn_rev	AGCTCGCCAATGGTGCTATGGG
**Primers for q-RTPCR**	
S.meliloti	
SM_xsc_QP_fwd	GTCATCGGCAACGCCATCCA
SM_xsc_QP_rev	TTGAGCCGCGTACCCAATGC
SM_sorT_QP_fwd	GCCGCTCATTTTCTGGTCACCC
SM_sorT_QP_rev	TCAACACGCCTCCGGAATCG
16S_Smel_qPCR_fwd	AGGCGGATTGTTAAGTGAGGGGTG
16S_Smel_qPCR_rev	TTTGCTCCCCACGCTTTC
SM_4050 QP-fwd	TCGAAACCGTGCTCAACGTCCG

SM_4050QP_rev	TTCACGCCGCCAATGCTC
SM_rpoEQP_fwd	TTCGCGATGCTCGGTGCTGA
SM_rpoEQP_rev	AGCCCGCATTTCGTGCATGG
SMc01420-21F	CCGACAACCGAGCCGTCTTGAT
SMc01420-21R	GGTGACGGAACTCTCCATCGCTTC
16sSmelQF100	GGTGAAGATAATGACGGTAACCGGAGA
16sSmelQR100	CCCAGTAATTCCGAACAACGCTAGC
SMc02156QF	CGGATGTCTGGACGAAGGAGACG
SMc02156QR	CGCAATGACTGCCGGACGAA
SMc04164QF100	AGCATTCGTCATCCGCATCGAGCC
SMc04164QR100	TCGTTGCGGTGCCGCTTCCA
SMc00821QF	GGCCGTCTGGCGCCTCTATCGAAAC
SMc00821QR	CGTGGCGCACTCCCCTGTAGAAGAG
SMc00108QF	GGTGGTGCATTTCCATGCCG
SMc00108QR	CGAGATGGTCGCCGAGCTTG
SMb21671QF	GCATCGCTTGGGAGAGGTGAAGGTCAA
SMb21671QR	TCGCCCATCAAGACTACGTGACGCA
S.novella	
SNQP16SF	TGGCAGACGGGTGAGTAACACGT
SNQP16SR	GCGGGTTCATCCAATGGCGA
SNsorAQP1F	AGCATTGATTGACGGCGACGA
SNsorAQP1R	ATCGACCCCGATGCATTCCG
SNsigEQP1F	AAAGCCCGCAAGATCCACCAGT
SNsigEQP1R	CGCCGAGCCGATCGAACATT
SNORF1QP1F	GTCATATGCGCGACGATGTCGG
SNORF1QP1R	GCGATCGCGATGGCCTGCAT
SN0991QF121N	TCACGCTGAAGGACATCGGCA
SN0991QR121N	CCGTTGGCGTTATGGGCGATA
SNRpoE1QF121N	CGAAAGGAGCCACCGTGAACC
SNRpoE1QR121N	TCCTGCACCAGATCGTCCGC
SN0993QF121N	GCCGAAGTGAGTGCCTATCTGGACG
SN0993QR121N	TCGGCACCGGCTCCTCGATCA
SN0994QF121N	GGCGGTCAATTTCTCCCTGCTTG
SN0994QR121N	ATCGACGCCCAGCCTTTGCAG
Sn0990QF121	CAGGAGATCGTCGTCGAGCAGC
SN0990QR121	TGGCCGAATTGTCGTGAGCG

Table S4 – sequences or species for phylogenetic trees – available as separate Excel file.

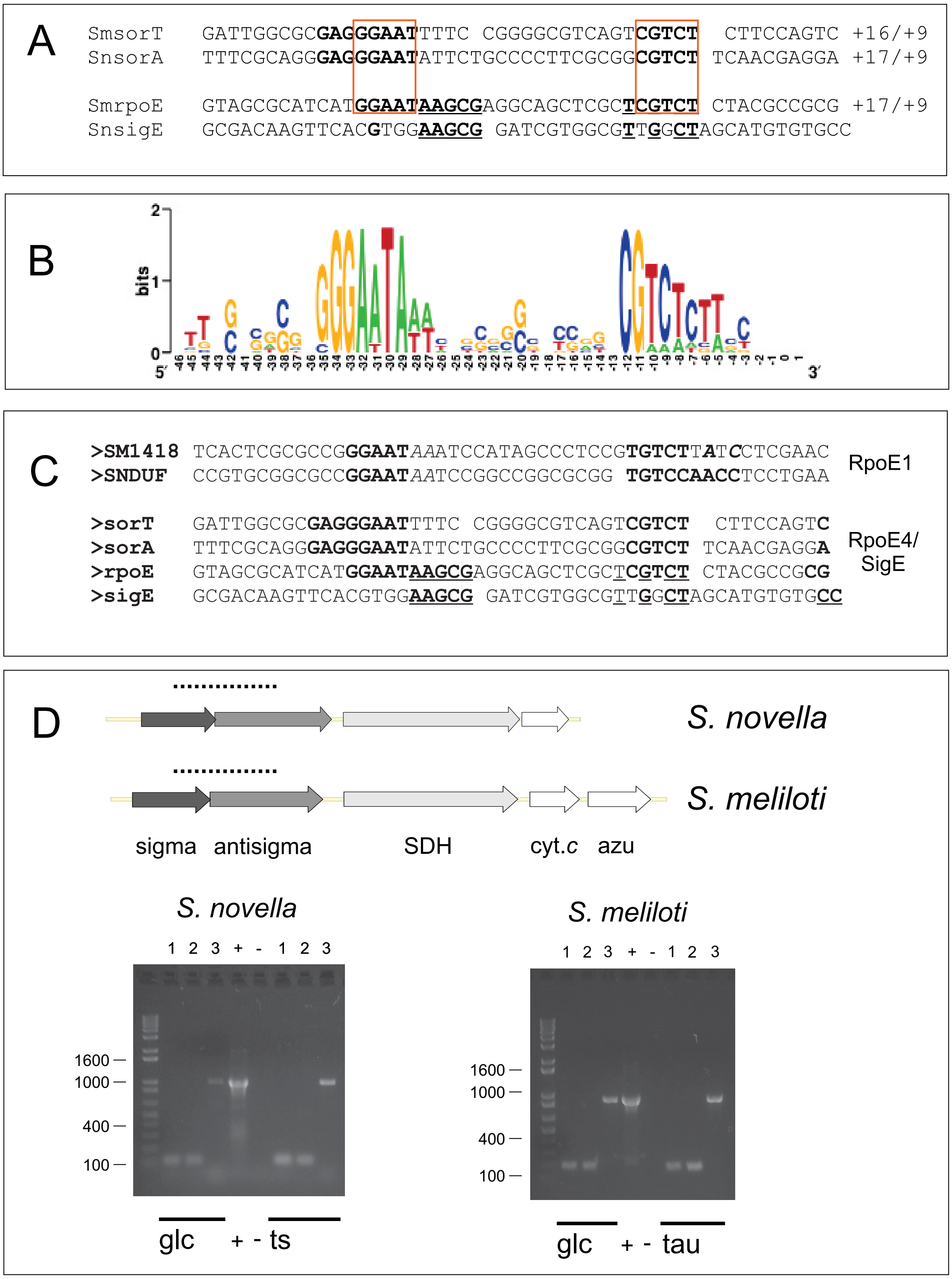

Figure S1 – Properties of the SOE encoding gene regions in *S. meliloti* and *S. novella*. **Panel A**: alignment of promoter consensus sequences predicted based on transcription start site mapping using 5'RACE. The Sn*sigE* promoter lacks the consensus found for Sn*sorA*, Sm*sorT* and Sm*rpoE4*. A unique sequence motif is shared between Sn*sigE* and Sm*rpoE4* promoter

regions. **Panel B**: Weblogo representation of the predicted promoter consensus. **Panel C**: Comparison of the promoter seuqences shown in Panel A with sections of the promoter regions of two RpoE1 controlled genes, Smc01418 and Sn*duf305* showing extensive similarities between the consensus sequences. **Panel D:** Co-transcription of ECF sigma factors with cognate antisigmafactor genes in *S. meliloti* and *S. novella*. Dotted lines represent the relative position of the PCR product generated. Both a schematic representation of the two operons as well as a PCR analysis carried out for two growth conditions for each strain is shown. Lane labels: **1** – 100 bp Sm*rpoE4* or Sn*sigE* PCR product amplified from cDNA **2** - 100 bp PCR product for the antisigma factor genes associated with Sm*rpoE4* or Sn*sigE* amplified from cDNA, **3** –PCR product bridging the ECF sigma factor and ASF genes (position indicated in operon schemes) amplified from cDNA, + - PCR product bridging the ECF sigma factor and ASF genes, amplified from gDNA, **-** - negative control – no template, primers for PCR product bridging the ECF sigma factor and ASF genes

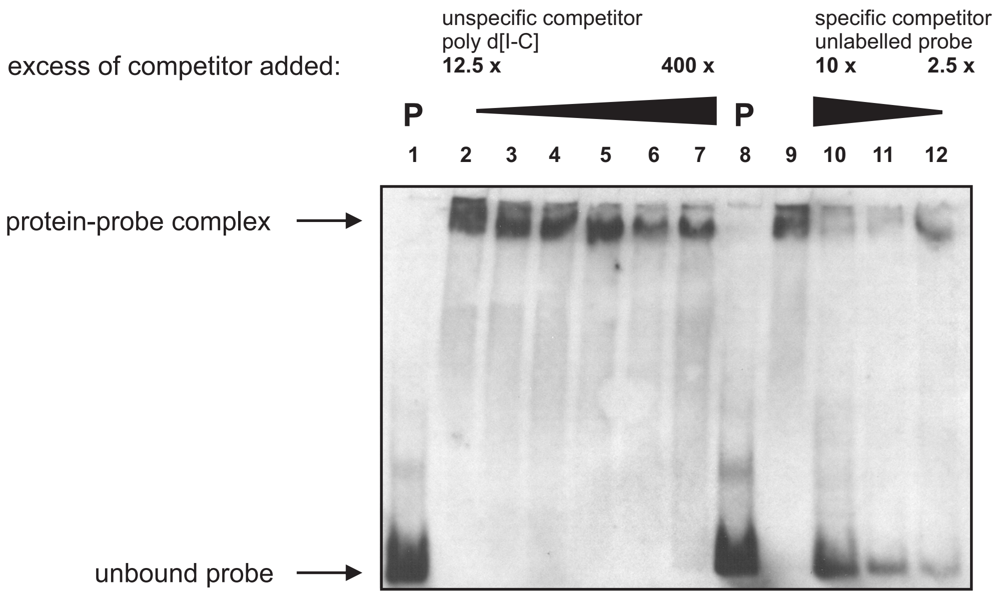

Figure S2 – EMSA assay demonstrating binding of purified *S. novella* SigE to a *sorA* promoter probe (120 bp). Binding reactions were separated on a 7.5% Polyacrylamide gel followed by electroblotting and non-radioactive detection (Dig Gelshift Kit 2, Roche) of the labelled DNA probe. Binding specificity was tested using either poly d[IC] as a non- specific competitor or unlabelled *sorA* probe as a specific competitor.

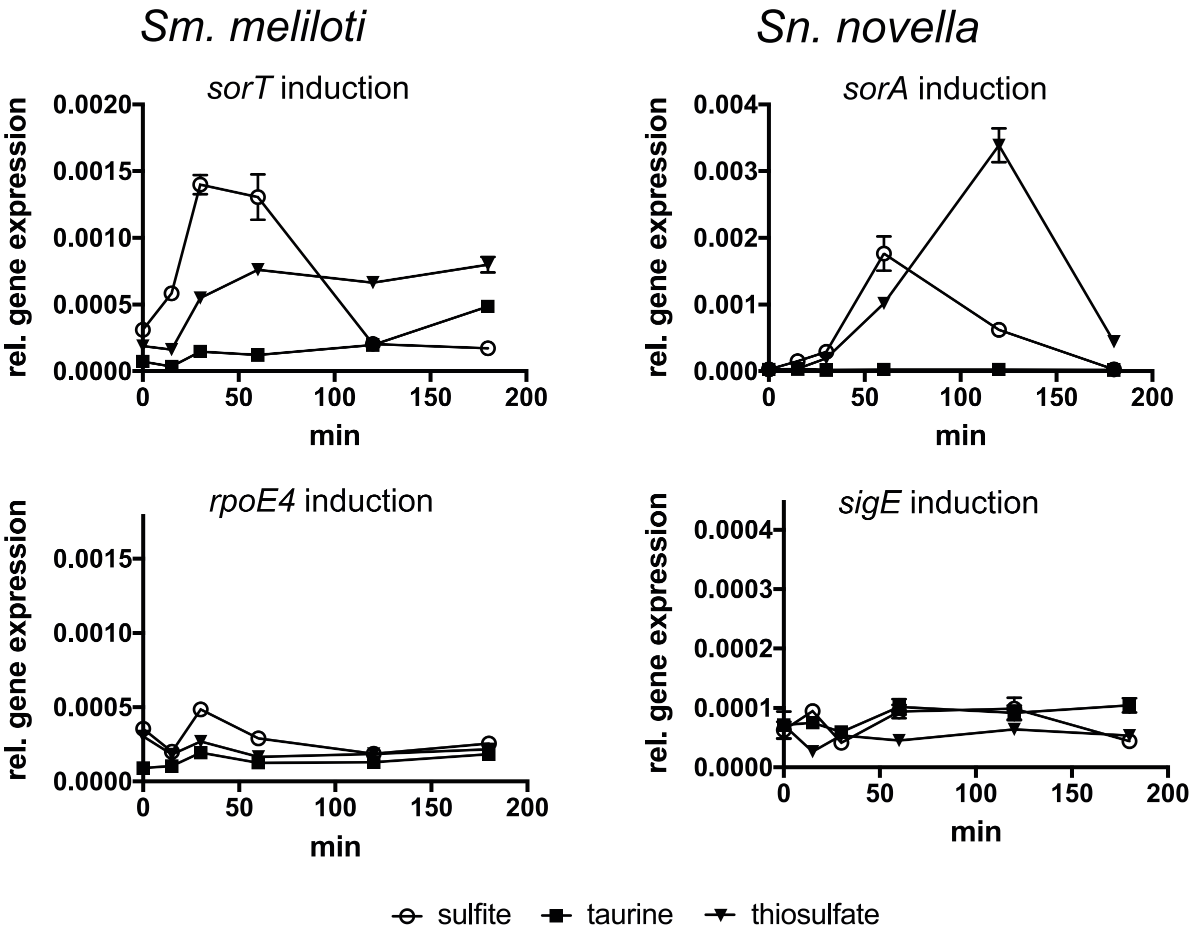

Figure S3 –SOE and ECF sigma factor gene expression following exposure to sulfite, taurine and thiosulfate in *Sm. meliloti* 1021 and *St. novella* 506^{T}. **Left:** *Sm. meliloti* gene expression induction, ***Top***: induction of the *sorT* SOE gene, ***Bottom***: induction of the *rpoE4* ECF sigma factor gene. **Right:** *St. novella* gene expression induction, ***Top***: induction of the *sorA* SOE gene, ***Bottom***: induction of the *rpoE4* ECF sigma factor gene. Data are shown as the average and standard deviation of at least three assays. 2-Way ANOVA of changes in rel. normalized gene expression compared to the t=0 value showed that for sulfite addition changes were statistically significant (alpha =0.05) from t=15 min (Sm) and t=30 min (Sn) with p= 0.0032 (Sn, 30 min) to <0.0001 for all other values, for thiosulfate from t=30 min (Sm) and t=60 min (Sn) with p <0.0001 for all datapoints, for taurine all values were not significant except Sm 120min p= 0.0232, and 180 min (p<0.0001). For clarity the p-values are not shown in the figure panes.

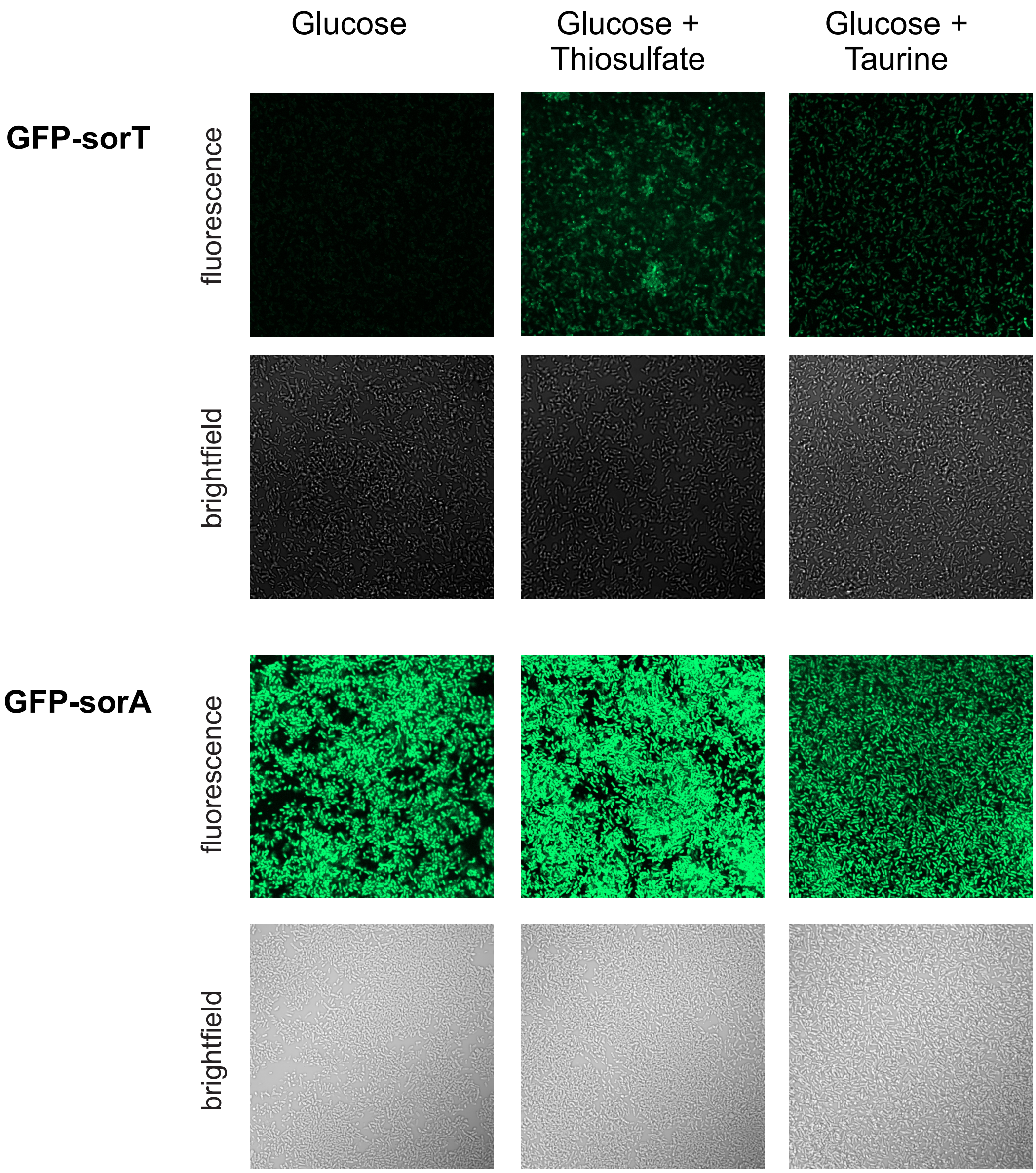

Figure S4 – Activity of GFP-fusions of SDH promoters (Sm*sorT* and Sn*sorA*) in *S. meliloti* 1021 grown with glucose (negative control no formation of sulfite), glucose plus thiosulfate (abiotic sulfite formation) or glucose and taurine (biotic sulfite formation). Both the fluorescence and brightfield images are shown. The constant activation of the of *sorA* promoter -GFP fusions in the *S. meliloti* background regardless of the growth substrate used is likely due

to interactions with SmRpoE1 and is in keeping with the proposed constant activation of SmRpoE1 in *S. meliloti* and the results from the beta galactosidase reporter gene assays that showed a strong induction of the *sorA* promoter by SmRpoE1.

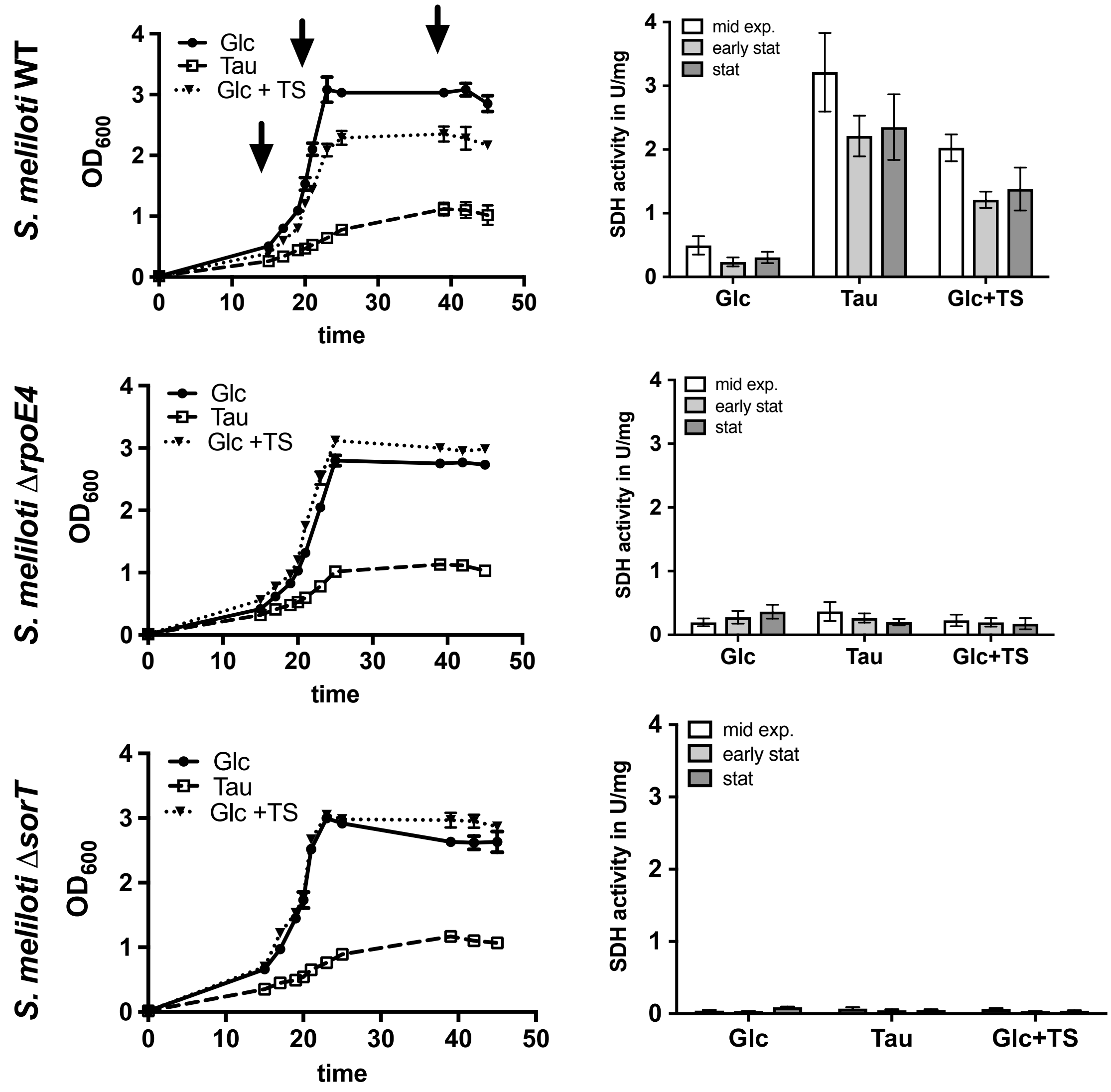

Figure S5 – Growth phase-dependent changes in SorT activity in *S. meliloti* ^{WT} (**Top**), *S. meliloti*$^{\Delta rpoE4}$ (**middle**) and Δ*sorT* strains (**bottom**). **Left** – *S. meliloti* growth on glucose, taurine and glucose & thiosulfate, arrows indicate sampling time-points. **Right** – Growth phase dependent changes in SorT activity in *S. meliloti*^{WT} on each of the three substrates at the times of sampling. Data shown are averages of three replicate determinations, errors shown are standard deviations.

Gene expression in *S. meliloti* WT and DrpoE4

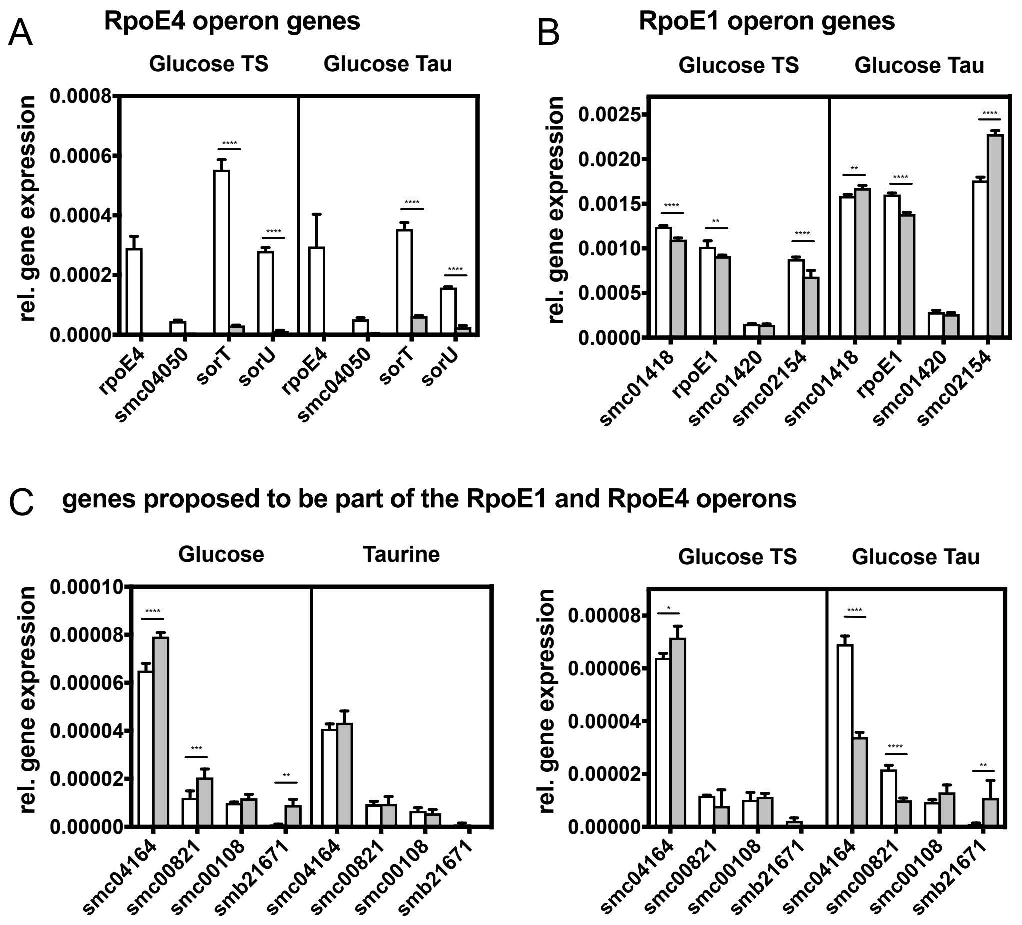

Figure S6 – Expression of *S. meliloti rpoE1* and *rpoE4* operon genes and accessory genes during growth of *S. meliloti* WT (white) and *S. meliloti* Δ*rpoE4* (grey) on glucose, taurine, glucose & thiosulfate or glucose & taurine. **Panel A**: expression of RpoE4 operon associated genes during growth on glucose + thiosulfate or glucose + taurine. **Panel B**: expression of RpoE1 operon associated genes during growth on glucose + thiosulfate or glucose + taurine. **Panels C** expression of genes proposed to be regulated by either RpoE4 and RpoE1 for all four conditions studied (RpoE1-*smc02154*; RpoE4 -*smc04164*, *smc00821* and *smc00108*). All data are shown as averages and standard deviations of at least three replicate determinations. 2 Way ANOVA was used to analyse the data, **** $p<0.0001$, ** $p=0.0012$-0.009.

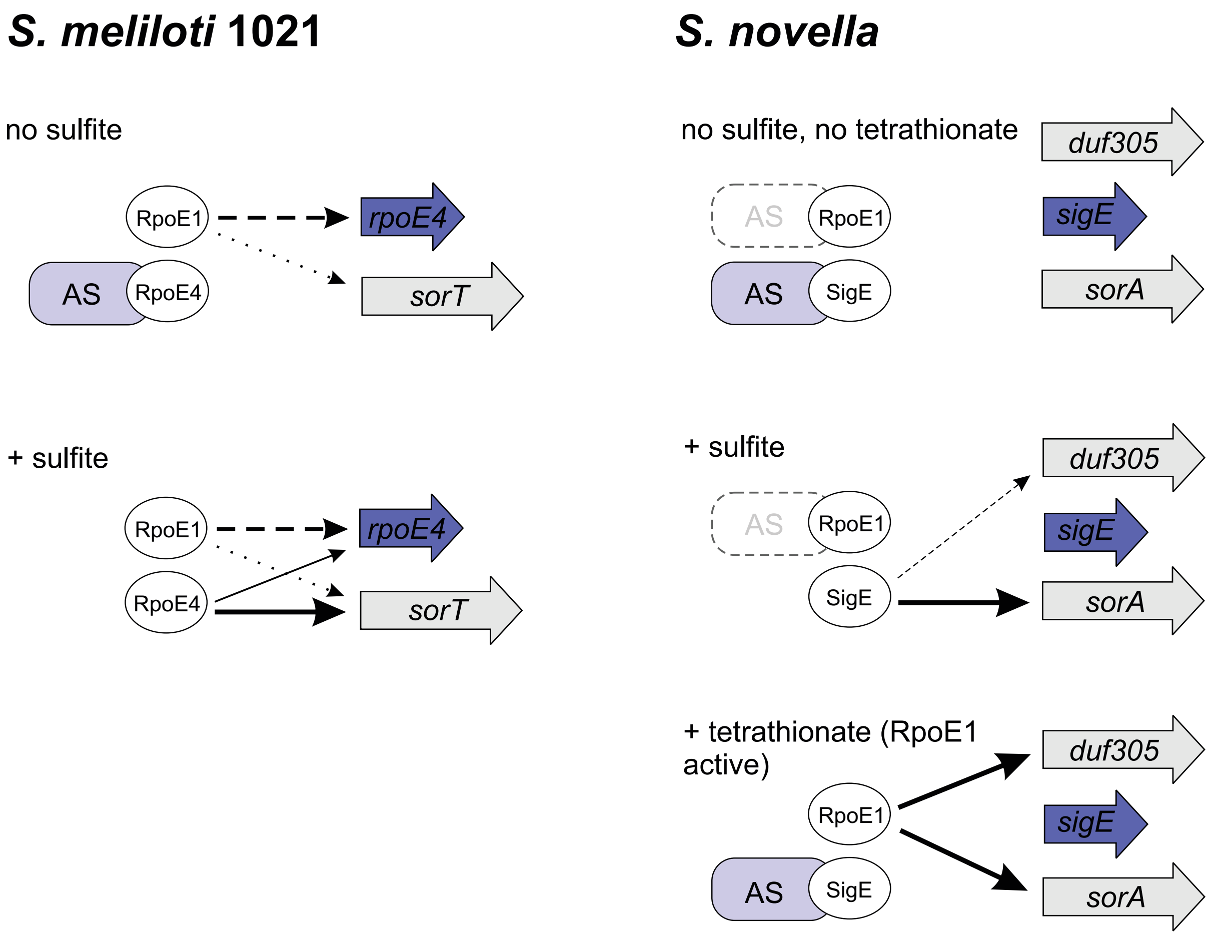

Figure S7 – Species specific models of the co-regulation of SOE gene expression by SmRpoE4/SnSigE and the respective RpoE1- like ECF sigma factors.

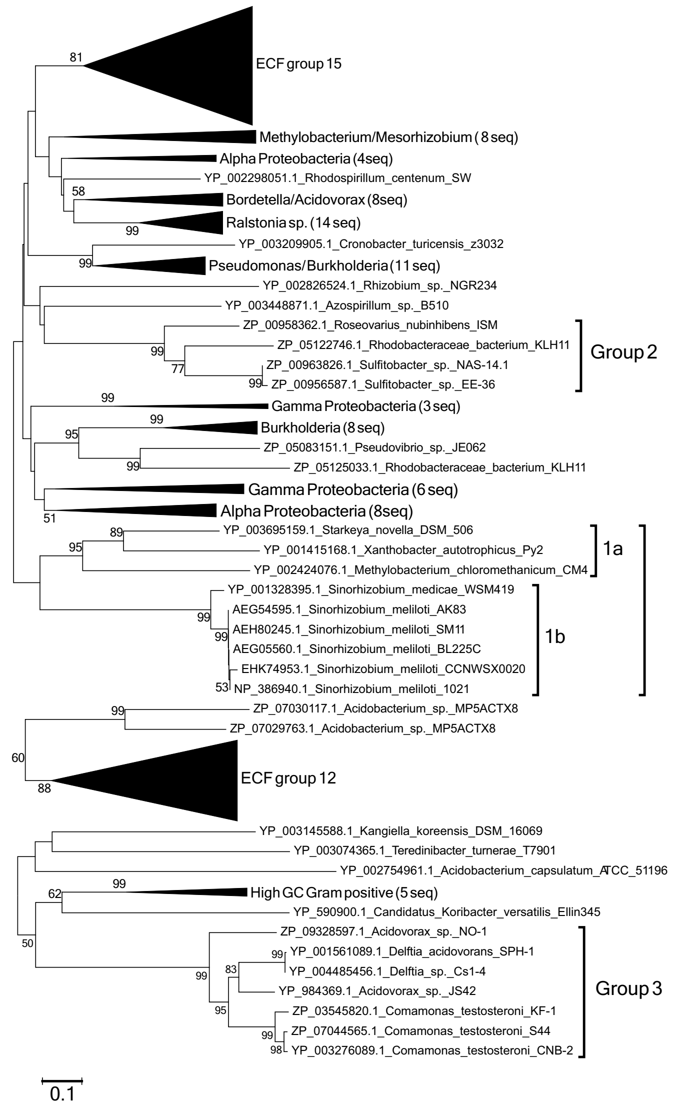

Figure S8 – Higher resolution image of the Phylogenetic tree shown in Figure 5. Please note that for the groups of SOE- associated sigma factors only a few, selected sequences are shown, Figure S9 shows a complete representation of these three groups.

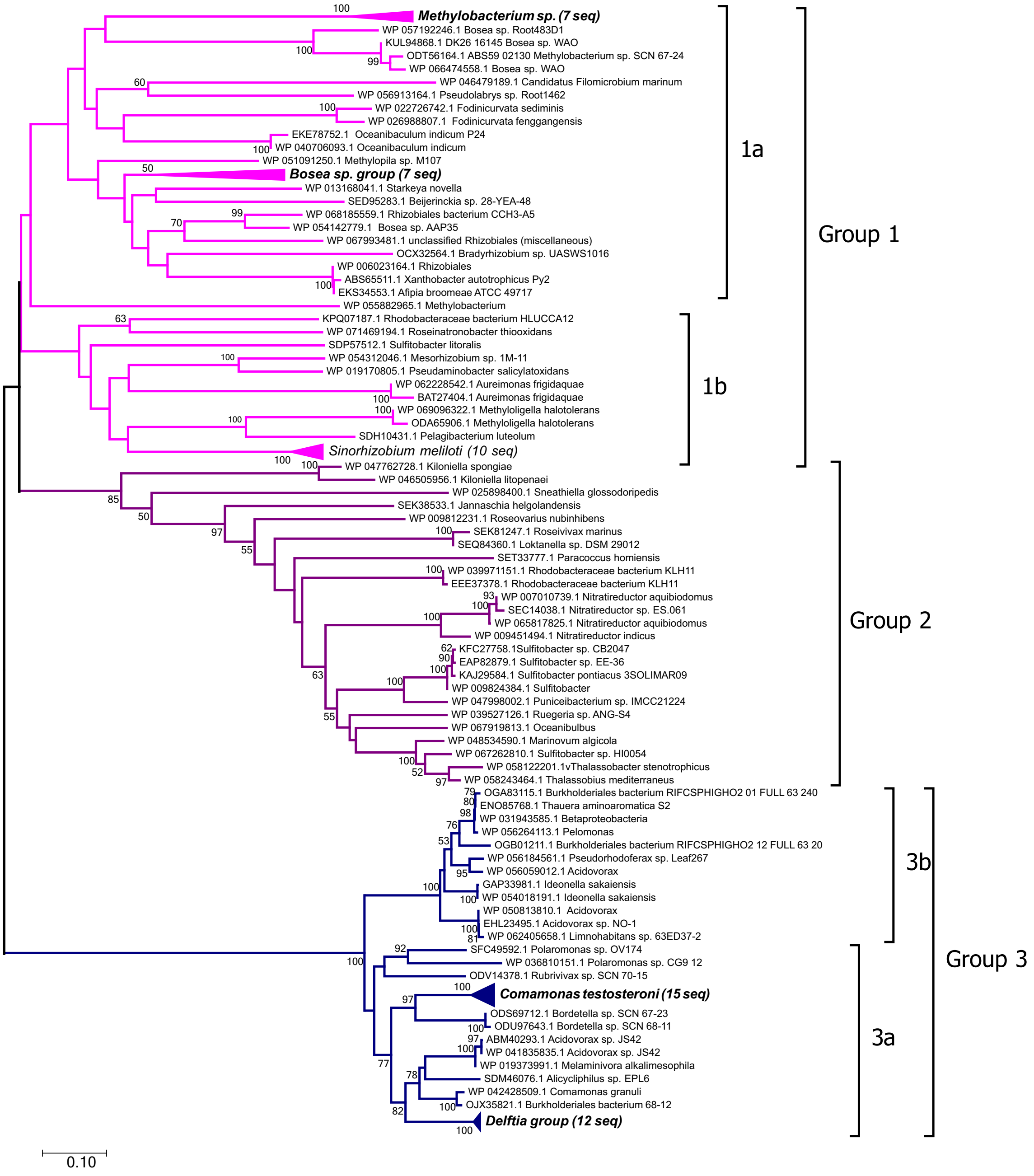

Figure S9 – Phylogenetic tree (Neighbor-joining) generated using only sequences of ECF sigmafactors associated with SOE genes. The three phylogenetic groups formed have been marked in colour. Robustness of the tree topology was tested using the bootstrap method.

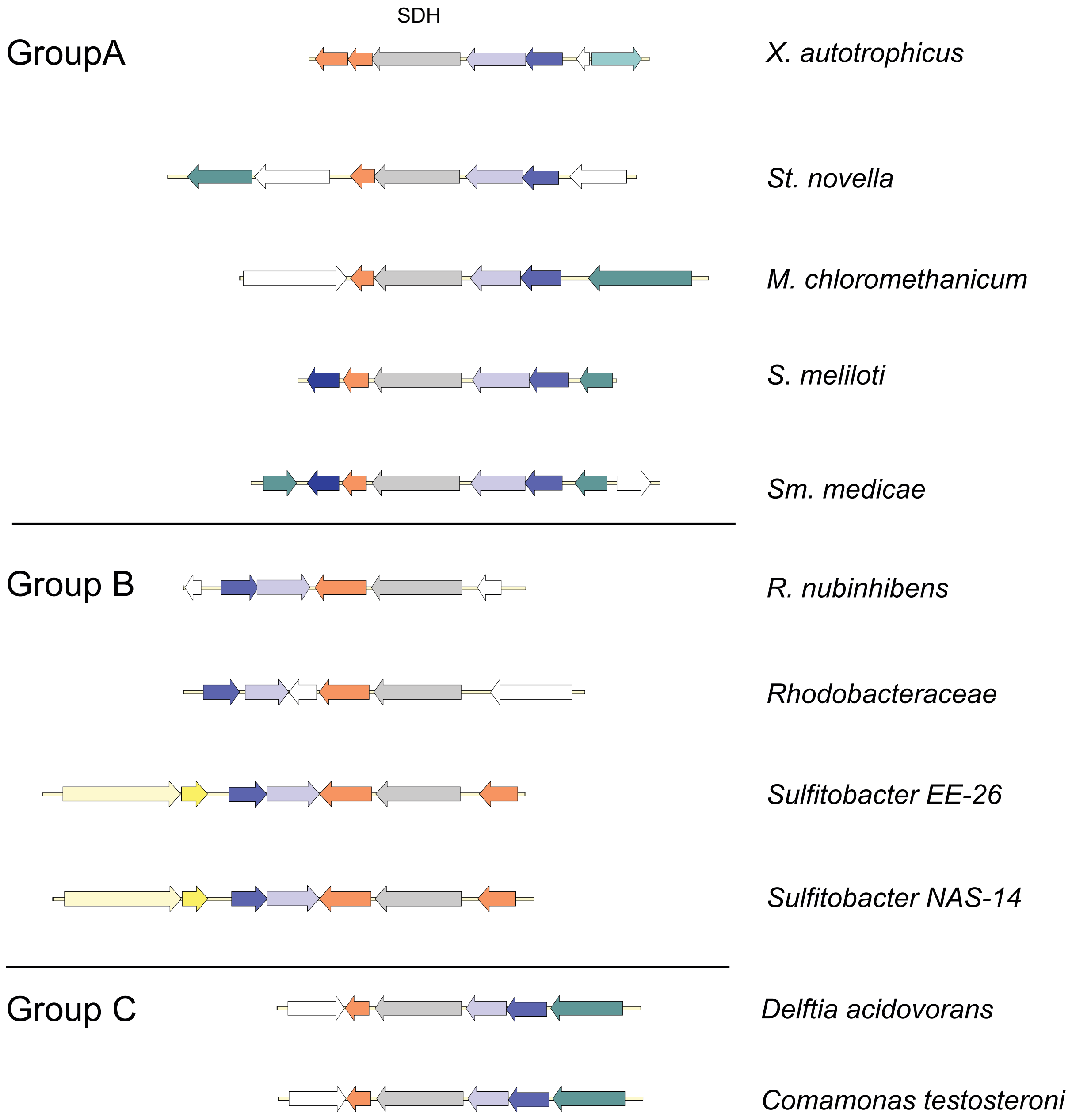

Figure S10 – Schematic representation of operon structures typically found in the three groups of SOE gene associated ECF sigmafactors. Colours: Dark blue – ECF sigmafactor, light blue – associated antisigmafactor, grey – SOE encoding gene, orange – cytochrome c encoding gene, purple – gene encoding azurin, green – gene encoding another type of transcriptional regulator, white – unrelated genes.
